# A new multilayer tree structure belief rule base-based prediction method for key indicators of flotation process

**DOI:** 10.1371/journal.pone.0336336

**Published:** 2026-02-02

**Authors:** Heng Dong, AoSheng Gong, Wei He

**Affiliations:** School of Computer Science and Information Engineering, Harbin Normal University, Harbin, Heilongjiang, China; Lodz University of Technology: Politechnika Lodzka, POLAND

## Abstract

The prediction of key indicators in the flotation process is crucial for optimizing operations, improving quality, and reducing consumption. However, indicator prediction itself suffers from complex nonlinear relationships, difficulties in model construction, and noise interference. To solve the above problems, this paper proposes a new model based on a multilayer tree structure belief rule base (MTS-BRB), termed MTS-BRB with attribute reliability (MTS-BRB-R). First, an initial prediction model is constructed using the MTS-BRB framework. Second, the attribute reliability is embedded into the model structure to enhance the robustness of its inference and prediction accuracy. Finally, the prediction of the tailings silica content in the iron ore flotation process is used as a case study to verify the effectiveness of the proposed model.

## 1 Introduction

The flotation process is an indispensable key step in the modern mining industry [1,2]. Its efficiency directly affects the comprehensive utilization of minerals. Improving flotation efficiency is a popular issue in the mining industry [3]. Realizing real-time detection of key indices in the flotation process is very important for improving flotation efficiency [4].

Currently, prediction methods for key indicators rely heavily on traditional data-driven modeling approaches. Many studies have explored this topic in the context of data-driven modeling [5,6]. For example, Zhang et al. [7] proposed a neural network model based on an encoder-decoder structure for predicting the tailings grade in the zinc flotation process. Nakhaei et al. [8] developed a prediction model for copper concentrate grade via artificial neural networks with process variables such as froth thickness, make-up water volume, and feed rate as inputs and provided guidance for production parameter optimization. A. Jiahedsaravani et al. [9] modeled the relationship between froth characteristics and performance parameters through image analysis and neural networks. Yuanyuan Pu et al. [10] developed a new deep learning network, FlotationNet, for predicting grade and recovery in the flotation process. Guichun He et al. [11] used a two-step approach, first using the box-and-line diagram method and filtering algorithm to address outliers and then using various modeling methods to predict flotation recovery. Fardis Nakhaei et al. [12] developed a recurrent neural network (RNN) model to predict copper flotation performance. The RNN achieved high accuracy, with correlation coefficients of 0.92 for grade and 0.9 for recovery, outperforming other compared models. Yousef Salehi et al. [13] proposed a vision model that uses an autoencoder for image inpainting and a multirate ARX model with image features to predict slow-rate process variables. The model employs the EM algorithm and a Kalman filter for parameter estimation and state smoothing, demonstrating effective fast-rate prediction of interface level in a separation cell. Shahbazi et al. [14] used a random forest algorithm to identify key variables such as bubble surface area flux, Reynolds number, and particle size, and successfully constructed a model for predicting flotation rate constants and recoveries with high accuracy. Tian et al. [15] designed a model predictive control (MPC) scheme for the nonlinear coupled partial differential-orthogonal differential equation (PADE) model of mineral flotation columns by means of a Cayley-Tustin time-discretization scheme. Tustin time discretization to address constraints and perturbations and achieve optimal control within the guaranteed steady-state output and constraints. Chen et al. [16] simulated the flotation process of chalcopyrite in a three-dimensional mineralized tube by coupling computational fluid dynamics (CFD) simulation with flotation dynamics models, including collision, attachment, and detachment probability models and bubble slip velocity models. Dong et al. [17] proposed a new optimization control method based on condition identification and multi model switching. Through the integration of fuzzy control, expert system and LS-SVM algorithm, the intelligent collaborative optimization of flotation column foam layer depth and reagent dosage was realized, providing an effective solution for intelligent control of complex industrial processes.

Data-driven modeling approaches rely heavily on the quality and scale of monitoring data [18]. Moreover, their predictive performance is largely constrained by the completeness and accuracy of the training samples. Although these models can handle high-dimensional data and achieve high prediction accuracy in specific scenarios, their modeling process usually lacks interpretability and requires high data volume, which leads to insufficient model reliability.

To address these inherent shortcomings of purely data-driven models, researchers have explored hybrid modeling approaches that fuse expert knowledge and data. Among them, Belief Rule Base (BRB) was proposed by Yang et al. [19]. As a hybrid-driven model, it can utilize both expert knowledge and data. In addition, as a rule-driven model, one of the significant advantages of the BRB model is its good interpretability, so BRB models are usually highly reliable. Owing to these advantages, BRBs have been widely used in the field of forecasting. However, traditional BRB models face the rule combination explosion problem when dealing with multi-parameter problems. The hierarchical BRB (H-BRB) model is a hierarchical model that can effectively alleviate the rule explosion problem [20]. However, during its construction, it relies on manually designing the model structure, which leads to a lack of self-organization ability and makes its reasonableness difficult to ensure.

To address the limitations of H-BRBs, Yang et al. [21] proposed a self-organized BRB with a tree hierarchical structure (MTS-BRB) and verified the effectiveness of the model by predicting the project risk. Gong et al. [22] used the MTS-BRB to predict the remaining battery life and proposed an interpretable optimization algorithm. The MTS-BRB performs well in terms of structural self-organization. Nevertheless, the traditional method does not fully consider disturbing factors in the input data during modeling, which makes the model susceptible to noise and compromises the stability and accuracy of its predictions.

Therefore, to solve the above problems, this paper introduces the concept of attribute reliability into the MTS-BRB model. A new prediction model, MTS-BRB with attribute reliability (MTS-BRB-R), is proposed to handle environmental noise through calculated reliability measures. The MTS-BRB-R model can provide more accurate and reliable predictions while modeling complex systems.

The main contributions of this paper are as follows:

(1) A reliable MTS-BRB model is proposed. Owing to the limitations of the BRB model, it can easily lead to a rule combination explosion when there are too many feature attributes. MTS-BRB, as a model based on the H-BRB, can address the multi-attribute rule explosion problem effectively. On this basis, the attribute reliability is derived and added to the model by analyzing the parameters to obtain the MTS-BRB-R model.

(2) Proposed iron ore flotation model based on the MTS-BRB-R. The model defines its initial parameters by incorporating expert knowledge. These parameters are subsequently adjusted through algorithm optimization and sample training. Finally, better prediction results can be achieved. Compared with other prediction methods, this method is more accurate, reliable, and flexible.

This paper is organized as follows. Sect 2 reviews the basics of the BRB model and defines the problem. Sect 3 first describes the construction of the MTS-BRB-based prediction model in detail. It then mechanistically analyzes the process of constructing and training the prediction model via the MTS-BRB-R approach. In Sect 4, simulation experiments are designed via a specific case study. A comparative study with other methods is then conducted to validate the proposed model. Finally, Sect 5 concludes the study.

## 2 Preliminaries and problem description

In Sect 2.1, the basics of BRBs are reviewed to provide the basis for the new prediction model proposed in this paper. In Sect 2.2, the research problem is described.

### 2.1 BRB Basics

In this section, the main elements of the MTS-BRB model are reviewed to provide a basis for proposing the new prediction model in this paper.

The BRB expert system consists of a series of belief rules. Usually, the first belief rule in a traditional BRB can be written as:

$$IF\left(X_{1}isA_{1}^{k}\right)\wedge \left(X_{2}isA_{2}^{k}\right)\wedge \cdots \wedge \left(X_{M}isA_{M}^{k}\right)\;Then\left\{\left(D_{1},\beta_{1,k}\right),\left(D_{2},\beta_{2,k}\right)\cdots \left(D_{N},\beta_{N,k}\right)\right\}\left(\sum_{n=1}^{N}\beta_{N,k}\leq 1\right)\;with\;rule\;weight\;\theta_{k}\left(k=1,2,\cdots ,L\right)\;and\;attribute\;weights\;\delta_{i}\left(i=1,2,\cdots ,M\right)$$ (1)

where $A_{i}^{k}$ is the reference value of the *i*th antecedent attribute in the *k*th rule. $\beta_{N,k}$ is a belief degree to which the consequent *D*_*N*_ is believed to be true. $\theta_{k}$ is the weight of the *k*th belief rule. $\delta_{i}$ is the weight of the *i*th attribute. *L* is the number of rules. *M* is the number of attributes.

To illustrate conventional BRB in detail, this paper takes key flotation process indicators as the prediction problem: the tailings silica content (namely *D*) is a function of slurry pH value (namely *X*_1_) and slurry density (namely *X*_2_). When there are three assessment ratings e.g. $\left\{A_{1,1},A_{1,2},A_{1,3}\}=\{A_{2,1},A_{2,2},A_{2,3}\}=\{\text{Low},\text{Middle},\text{High}\right\}$, provided for *X*_1_ and *X*_2_, and three consequents, e.g. $\left\{D_{1},D_{2},D_{3}\}=\{\text{High},\text{Medium},\text{Low}\right\}$, provided for *D* by domain expert, a conventional BRB can then be constructed by covering all possible combinations of each assessment rating for all antecedent attributes, as shown in Table 1.

**Table 1:** Example of a conventional BRB for flotation process metric prediction.

Rule No.	Weight *θ*	pH $X_{1}$		∧	Density $X_{2}$		Content *D*		
		Val	Level		Val	Level	High	Mid	Low
*R*_1_	0.9	$A_{1}^{1}$	L	∧	$A_{2}^{1}$	L	0.6	0.3	0.0
*R*_2_	0.1	$A_{1}^{2}$	L	∧	$A_{2}^{2}$	M	0.5	0.5	0.0
*R*_3_	0.5	$A_{1}^{3}$	L	∧	$A_{2}^{3}$	H	0.7	0.3	0.0
*R*_4_	0.8	$A_{1}^{4}$	M	∧	$A_{2}^{4}$	L	0.3	0.5	0.2
*R*_5_	0.3	$A_{1}^{5}$	M	∧	$A_{2}^{5}$	M	0.2	0.5	0.3
*R*_6_	0.2	$A_{1}^{6}$	M	∧	$A_{2}^{6}$	H	0.1	0.9	0.0
*R*_7_	1.0	$A_{1}^{7}$	H	∧	$A_{2}^{7}$	L	0.0	0.3	0.7
*R*_8_	0.1	$A_{1}^{8}$	H	∧	$A_{2}^{8}$	M	0.0	0.2	0.8
*R*_9_	0.6	$A_{1}^{9}$	H	∧	$A_{2}^{9}$	H	0.0	0.1	0.9

From Table 1, it can be found that a belief rule such as *R*_1_ contains that when pH value is Low and slurry density is Low, then 60% sure that the tailings silica content is High and 30% is Medium. The weight of *R*_7_ is 1.0, indicating the importance of *R*_7_ over other rules. The weight of slurry pH and slurry density is 0.7 and 0.9, respectively, indicating the different importance of these two attributes. where $A_{n}^{m}$ denotes the *n*th antecedent reference attribute for the *m*th rule. $\beta_{N,K}$ denotes the belief degree of the *K*th rule for the *N*th result.

A key limitation of the traditional BRB is that it involves generating an exponential number of combinations from all assessment ratings [23]. Specifically, when there are M antecedent attributes and each has N reference values, the rule base size becomes *N*^*M*^. This finding demonstrates that the size of the conventional BRB model is exponentially related to the number of antecedent attributes and their assessment ratings. Consequently, any increase in these numbers causes the rule base to grow exponentially, leading to the rule explosion problem.

### 2.2 Problem description

This section formulates the problem.

**Problem 1:** In the traditional BRB, the number of rules grows exponentially with the increasing number of features M. Assuming that there are three assessment levels, the relationship between the number of rules and the number of features is shown in the following equation: *rule* = 3^*M*^. Multi-parameter problems suffer not only from rule explosion but also from model construction. Taking the traditional hierarchical model as an example, ordinary hierarchical structures tend to delineate levels on the basis of empirical experience or multiple statistical data. This method lacks a set of complex and standard modeling processes. Therefore, a reasonable construction method is needed to construct hierarchical models.

**Problem 2:** In BRB, attributes are used as inputs to the model, and their reliability directly affects the reliability of the model. Using unreliable attributes may produce unreliable model outputs. Therefore, it is necessary to improve the existing model to increase its reliability and accuracy.

## 3 Proposed MTS-BRB-R modeling and training process

**Fig 1:**
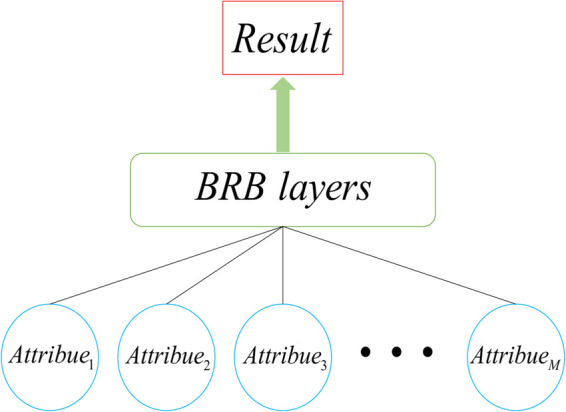
Structure of the H-BRB model.

The H-BRB is a method designed to address the combinatorial rule explosion problem. It employs a hierarchical design that decomposes the complex decision-making process of a system into multiple levels, each dedicated to solving specific subproblems, thereby effectively alleviating the issue. This approach emphasizes incorporating all the attributes. It builds them into a large-scale, expert-knowledge-driven model in a top-down manner. This allows all attributes to function within a single BRB structure, as illustrated in Fig 1.

Although the H-BRB performs well in handling complexity and uncertainty, it lacks a standardized methodology for selecting attributes to use as inputs for its sub-models. This shortcoming leads to the following problems in the modeling process: first, the modeler must rely on subjective experience and domain knowledge to filter the attributes, which not only increases the complexity and time cost of modeling but also may lead to unstable model performance; second, owing to the lack of standardized selection guidelines, the consistency and repeatability of the H-BRB model across different domains and application scenarios are difficult to ensure.

**Fig 2:**
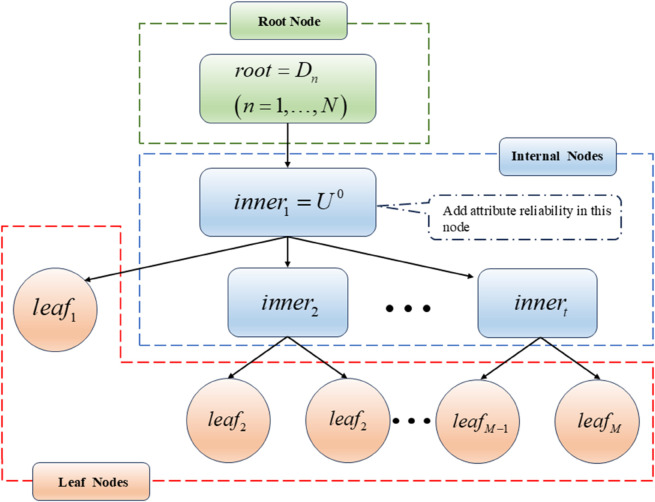
Multilayer tree BRB model.

To solve the above problems, Yang et al. [21] designed a multilayer tree structure to provide an effective guidance scheme for H-BRB modeling. The MTS-BRB model structure is shown in Fig 2.

The MTS-BRB consists of three types of nodes: root nodes, internal nodes, and leaf nodes. Root nodes: Nodes with no parent nodes become root nodes, and the root node consists of the set of all the resultant attributes. Internal nodes: Nodes with parent and child nodes become internal nodes, and each internal node can be interpreted as a child BRB containing the next level of leaf nodes or internal nodes. Leaf nodes: Nodes without children become leaf nodes, and each leaf node corresponds to a feature attribute.

However, since MTS-BRB uses attributes as inputs to the model, their reliability directly affects the reliability of the model. To address the impact of attribute unreliability on the model, this paper introduces attribute reliability within the BRB structure, which is composed of the first internal node. The location of its introduction is shown in Fig 2.

The modeling process of the MTS-BRB-R can be divided into three steps: the construction of the multilayer tree structure (MTS), the calculation of attribute reliability, and the construction of the BRB. The construction of the MTS is divided into three parts: determining the root node, determining the leaf nodes, and generating the internal nodes. The BRB model is first constructed by establishing a rule base with expert knowledge. Its inference process then involves four stages: (1) converting input data, (2) calculating rule activation weights, (3) integrating rules to update belief degrees, and (4) aggregating the results to produce a final output.

### 3.1 MTS-BRB-R modeling process

The process of MTS is described in detail in Part A, the calculation method of attribute reliability is described in Part B, and the specific process of generating the corresponding MTS-BRB-R system on the basis of the MTS tree structure is described in Part C.

#### Part A: MTS structure construction.

Suppose that the problem to be modeled is described as $f(X_{1},\ldots ,X_{m})=D$, where *X*_*m*_ denotes the *m*th antecedent attribute. *D* denotes the consequent attribute. The model is built upon *M* antecedent attributes, where the *m*th attribute is characterized by *J*_*m*_ discrete reference values $A_{m,1},A_{m,2},\ldots ,A_{m,J_{m}}$. These reference values, known as attribute references, define the possible states for each attribute. The formula for calculating the attribute correlation is as follows [21,24].

$$AR\left(X_{m},X_{n}\right)=\frac{I\left(X_{m},X_{n}\right)}{H\left(X_{m},X_{n}\right)}$$ (2)

where $AR\left(X_{m},X_{n}\right)$ denotes the correlation between the antecedent attributes *X*_*m*_ and *X*_*n*_, which can be understood as the proportion of uncertainty reduction of *X*_*m*_ after *X*_*n*_ is known. Therefore, the smaller the AR value between the attributes is, the greater the independence of the two and the lower the correlation. $I\left(X_{m},X_{n}\right)$ denotes the mutual information and $H\left(X_{m},X_{n}\right)$ denotes the entropy of the two, which is obtained via the Eqs (3)–(4).

$$H\left(X_{m},X_{n}\right)=-\sum_{i=1}^{J_{m}}\sum_{j=1}^{J_{n}}P_{m,n}\left(X_{m}\text{ is }A_{m,i}\wedge X_{n}\text{ is }A_{n,j}\right)\;\times \log P_{m,n}\left(X_{m}\text{ is }A_{m,i}\wedge X_{n}\text{ is }A_{n,j}\right)$$ (3)

$$I\left(X_{m},X_{n}\right)=\sum_{i=1}^{J_{m}}\sum_{j=1}^{J_{n}}P_{m,n}\left(X_{m}\text{ is }A_{m,i}\wedge X_{n}\text{ is }A_{n,j}\right)\;\times \log\frac{P_{m,n}\left(X_{m}\text{ is }A_{m,i}\wedge X_{n}\text{ is }A_{n,j}\right)}{P_{m}\left(X_{m}\text{ is }A_{m,i}\right)\times P_{n}\left(X_{n}\text{ is }A_{n,j}\right)}$$ (4)

where $P_{m,n}\left(X_{m}\text{ is }A_{m,i}\wedge X_{n}\text{ is }A_{n,j}\right)$ denotes the probability that attribute *X*_*m*_ is equal to the reference value *A*_*m*,*i*_ and attribute *X*_*n*_ is equal to the reference value *A*_*n*,*j*_; similarly, $P_{m}\left(X_{m}\text{ is }A_{m,i}\right)$ denotes the probability that attribute *X*_*m*_ is equal to the reference value *A*_*m*,*i*_. Their calculation process is described below.

**Step 1:** Determine the root node In the MTS, the root node is set as the consequent attribute *root* = *D*. The root node is the only node without a parent.

**Step 2:** Identify the first internal node The first internal node *inner*_1_ consists of the antecedent attribute $U^{0}=\{X_{1},\ldots ,X_{M}\}$. The first internal node contains all nodes except the root node.

**Step 3:** Generate leaf nodes with the remaining internal nodes

**Step 3.1:** specify the cluster size *ε*

The cluster size determines the number of elements of any internal node. The internal node elements include two kinds of elements: leaf nodes $leaf=\{X_{m}\}(m=1,\ldots ,M)$ and internal nodes: *inner* = *U*^*t*^.

**Step 3.2:** Calculate the AR between each attribute

Assume that each antecedent attribute *X*_*m*_ has an attribute reference value *A*_*m*,*j*_ with a utility value of $u\left(A_{m,j}\right)\left(j=1,\ldots ,J_{m}\right)$. When there are *T* inputs $x_{t}=\left(x_{1,t},\ldots ,x_{m,t}\right)$ where (t=1,…,T), the following belief distribution can be obtained via a technique that is based on equivalent changes in utility:

$$S\left(x_{t},X_{m}\right)=\left\{\left(A_{m,j},\alpha_{m,j}^{t}\right);j=1,\ldots ,J_{m}\right\}$$ (5)

where $\alpha_{m,j}^{t}$ denotes the belief degree of the reference level *A*_*m*,*j*_ on the antecedent attribute *X*_*m*_. $\alpha_{m,j}^{t}$ can be obtained from the Eq (6).

$$\alpha_{m,j}^{t}=\frac{u\left(A_{m,j+1}\right)-x_{m,t}}{u\left(A_{m,j+1}\right)-u\left(A_{m,j}\right)}\;\text{and}\;\alpha_{m,j+1}^{t}=1-\alpha_{m,j}^{t},\;\text{if}\;u\left(A_{m,j}\right)\leq x_{t,m}\leq u\left(A_{m,j+1}\right)$$ (6)

On the basis of the Eqs (5)–(6), $P_{m,n}(X_{m}\text{ is }A_{m,i}\wedge X_{n}\text{ is }A_{n,j})$ and $P_{m}(X_{m}\text{ is }A_{m,i})$ can be obtained via the Eqs (7)–(8).

$$P_{m,n}\left(X_{m}\text{ is }A_{m,i}\wedge X_{n}\text{ is }A_{n,j}\right)=\frac{\sum_{t=1}^{T}\alpha_{m,i}^{t}\times \alpha_{n,j}^{t}}{T}$$ (7)

$$P_{m}\left(X_{m}\text{ is }A_{m,i}\right)=\frac{\sum_{t=1}^{T}\alpha_{m,i}^{t}}{T}$$ (8)

Finally, the two are brought into Eqs (3) and (4) to calculate the AR values obtained. The table of AR values obtained is shown in Table 2.

**Table 2:** AR values.

*AR*	*X*_1_	*X*_2_	*X*_*m*_	*X*_*M*_
*X*_1_	1	$AR(X_{1},X_{2})$	$AR(X_{1},X_{m})$	$AR(X_{1},X_{M})$
*X*_2_	$AR(X_{2},X_{1})$	1	$AR(X_{2},X_{m})$	$AR(X_{2},X_{M})$
*X*_*m*_	$AR(X_{m},X_{1})$	$AR(X_{m},X_{2})$	1	$AR(X_{m},X_{M})$
*X*_*M*_	$AR(X_{M},X_{1})$	$AR(X_{M},X_{2})$	$AR(X_{M},X_{m})$	1

As shown in Table 2, the AR value of any item with itself is 1. This result indicates that the knowledge of the item’s presence reduces the uncertainty about its own presence by 100%, which is a logically inevitable and perfect association.

**Step 3.3:** Hierarchical clustering of antecedent attributes based on correlation between attributes

In Step 2, the first internal node *inner*_1_ is $U^{0}=\{X_{1},\ldots ,X_{M}\}$, where *M* denotes the number of antecedent attributes in the problem. The *ε* pivot elements are generated according to the cluster size, and the *ε* pivot elements are chosen by Eq (9).

$$P_{t}=\left\{ \begin{array}{cc} \arg\min_{X_{m}\in U^{0}}\left\{\sum_{X_{n}\in U^{0}}AR\left(X_{m},X_{n}\right)\right\}, & \text{if }t=1\\ \arg\min_{X_{m}\in U^{0}-\left\{P_{1},\ldots ,P_{t-1}\right\}}\left\{\sum_{X_{n}\in \left\{P_{1},\ldots ,P_{t-1}\right\}}AR\left(X_{m},X_{n}\right)\right\}, & \text{otherwise} \end{array}\right.$$ (9)

The Eq (9) shows that the pivot point element *P*_*t*_ has minimal correlation with the elements within $U^{0}-\{P_{1},\ldots ,P_{t-1}\}$.

Next, each pivot point element is used to generate a subset of attributes $Sub\text{-}U^{t}$, both $Sub\text{-}U^{t}=\{P_{t}\}$.

Then, all the elements $X_{m}(X_{m}\in U^{0})$ are assigned to the *ε* subsets via the following assignment process:

$$Sub\text{-}U^{t}=\{X_{m}\}\cup Sub\text{-}U^{t},\;t=\arg\max_{n=1,\ldots ,\varepsilon }\left\{AR\left(P_{n},X_{m}\right)\right\}$$ (10)

For each subset $Sub\text{-}U^{t}$, its new pivot point $P_{t}^{\prime }$ should be selected via the Eq (11). If $\left\{P_{t}^{\prime };t=1,\ldots ,\varepsilon \right\}$ is exactly equal to $\left\{P_{t};t=1,\ldots ,\varepsilon \right\}$, then the clustering process is complete. When the number of elements in any subset is greater than the cluster size $\left|Sub\text{-}U^{t}\right|>\varepsilon$, this subset is considered as *U*^0^ and re-execute Step 2. If at the end of clustering, there is a subset with the number of elements equal to 1, this subset is considered a leaf node.

$$P_{t}^{\prime }=\arg\max_{X_{m}\in Sub\text{-}U^{t}}\left\{\sum_{X_{n}\in Sub\text{-}U^{t}}AR\left(X_{m},X_{n}\right)\right\}$$ (11)

**Fig 3:**
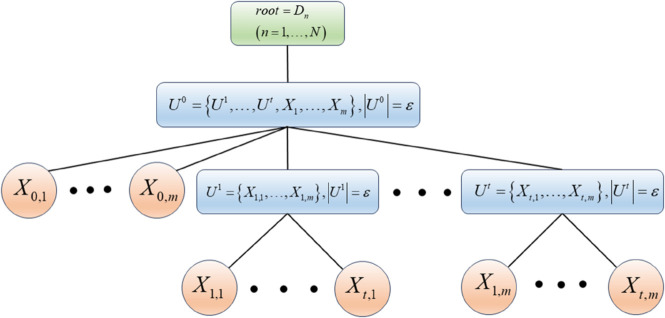
Structure of the MTS model.

The final constructed structure is shown in Fig 3.

As shown in Fig 3, each internal node in the structure contains *ε* elements. The first internal node encompasses all other internal nodes and all leaf nodes. The elements contained within an internal node can be one of two types: (1) other internal nodes, (2) leaf nodes. Leaf nodes are terminal elements that are defined by a set of attributes.

#### Part B: Calculating attribute reliability.

In engineering practice, the observation data collected by sensors may fluctuate due to interference factors. When the observation data fluctuate greatly, the current system is considered unreliable [25]. Therefore, unreliable observation data can be selected according to the tolerance range. If the observation data are out of the tolerance range, they do not represent the correct system information and are unreliable.

Suppose the input to the *i*th antecedent attribute *X*_*i*_ is $\{X_{i}(j){\}}_{j=1}^{T_{i}}$. *T*_*i*_ denotes the number of input data for the antecedent attribute *X*_*i*_. *ψ* is the expert-determined tolerance range. $\overset{―}{X_{i}}$ and $\sigma_{i}$ represent the mean and standard deviation of the *i*th antecedent attribute observation, respectively.

The attribute reliability *r*_*i*_ of the *i*th antecedent attribute is subsequently calculated via the Eq (12).

$$X_{i}(j)\;(i=1,2,\ldots ,M)\;(j=1,2,\ldots ,T_{i})\;y_{i,j}=\left\{ \begin{array}{cc} 0 & \overset{―}{X_{i}}-\psi \sigma_{i}\leq X_{i}(j)\leq \overset{―}{X_{i}}+\psi \sigma_{i}\\ 1 & \text{otherwise} \end{array}\right.\;count_{i}=\sum_{j=1}^{T_{i}}y_{i,j}\;r_{i}=\frac{T_{i}-count_{i}}{T_{i}}$$ (12)

where *r*_*i*_ denotes the reliability of the *i*th antecedent attribute. *M* represents the number of antecedent attributes. In particular, for the consequent attribute output by the sub-BRB, the default is fully reliable, which means that the consequent attribute reliability of the sub-BRB is 1.

#### Part C: BRB model structure construction.

After obtaining the MTS in part A, a BRB model is constructed for each internal node. It is worth noting that attribute reliability is incorporated only into the first internal node during the construction of the BRB models for internal nodes, as this node is responsible for training the overall model. The remaining internal nodes are constructed following the standard BRB modeling approach.

The initial parameters of the BRB model including rule weights, attribute weights, and belief degrees are determined by domain experts leveraging extensive operational experience and historical data from the flotation process. Specifically, rule weights are designed to reflect the relative importance of different operating conditions, attribute weights represent the influence of each process variable, and belief degrees characterize uncertain relationships between inputs and outputs. This expert-driven initialization not only incorporates domain knowledge but also provides a physically meaningful starting point for subsequent parameter optimization.

**Step 1:** Calculate attribute reliability

After constructing the MTS, the corresponding attribute reliability can be calculated using the method described in Part B.

**Step 2:** Calculate the match between the input data sample and the belief rule

For input *x*_*i*_, its match $\alpha_{i}^{j}$ to the attribute reference is obtained via Eq (13).

$$\alpha_{i}^{j}=\left\{ \begin{array}{cc} \frac{A_{i,k}-x_{i}}{A_{i,k+1}-A_{i,k}}, & j=k,\;A_{i,k}\leq x_{i}\leq A_{i,k+1}\\ 1-\alpha_{i}^{j}, & j=k+1\\ 0, & j=1,2,\ldots ,K_{i},\;j\neq k,k+1 \end{array}\right.$$ (13)

where *x*_*i*_ denotes the input data for the *i*th antecedent attribute. *A*_*i*,*k*_ and *A*_*i*,*k* + 1_ denote the reference values of the *i*th antecedent attribute within the [k,k  +  1] rule. *K*_*i*_ denotes the number of rules containing the *i*th antecedent attribute within the whole system.

**Step 3:** Aggregate attribute reliability and attribute weights

The formula for aggregating attribute reliability and attribute weights is as follows.

$$C_{i}=\left\{ \begin{array}{cc} \overset{―}{\delta_{i}} & \text{otherwise}\\ \frac{\overset{―}{\delta_{i}}}{1+\overset{―}{\delta_{i}}-r_{i}} & \text{when }inner_{i}=inner_{1} \end{array}\right.\;\text{where}\;\overset{―}{\delta_{i}}=\frac{\delta_{i}}{\max_{i=1,\ldots ,M}\left\{\delta_{i}\right\}},\;0\leq \overset{―}{\delta_{i}}\leq 1$$ (14)

where *C*_*i*_ denotes the combination parameter that considers both attribute weights and attribute reliability. *r*_*i*_ denotes the reliability of the *i*th attribute. $\delta_{i}$ denotes the weight of the *i*th attribute. $\overset{―}{\delta_{i}}$ denotes the relative weight of the *i*th attribute. When the attribute is completely reliable *r*_*i*_ = 1, then $C_{i}=\overset{―}{\delta_{i}}$.

**Step 4:** Calculate rule activation weights

According to the calculated combination parameter *C*_*i*_, which considers both attribute weights and attribute reliability, the formula for calculating the rule activation weights is as follows:

$$\omega_{k}=\frac{\theta_{k}\prod_{i=1}^{M}{\left(\alpha_{i}^{k}\right)}^{C_{i}}}{\sum_{l=1}^{L}\theta_{l}\left(\prod_{i=1}^{M}{\left(\alpha_{i}^{l}\right)}^{C_{i}}\right)},\;k=\{1,\ldots ,L\}$$ (15)

**Step 5:** Generate belief degree

The belief degree in the results ${\hat{\beta }}_{n}(n=1,\ldots ,N)$ is generated by the parsing ER algorithm fused with the activation rule, which is calculated via Eqs (16) and (17).

$${\hat{\beta }}_{n}=\frac{\eta \times \left[\prod_{k=1}^{L}\left(\omega_{k}\beta_{n,k}+1-\omega_{k}\sum_{i=1}^{N}\beta_{i,k}\right)-\prod_{k=1}^{L}\left(1-\omega_{k}\sum_{i=1}^{N}\beta_{i,k}\right)\right]}{1-\eta \times \left[\prod_{k=1}^{L}\left(1-\omega_{k}\right)\right]}$$ (16)

$$\eta ={\left[\sum_{j=1}^{N}\prod_{k=1}^{L}\left(\omega_{k}\beta_{j,k}+1-\omega_{k}\sum_{i=1}^{N}\beta_{i,k}\right)-\left(N-1\right)\prod_{k=1}^{L}\left(1-\omega_{k}\sum_{i=1}^{N}\beta_{i,k}\right)\right]}^{-1}$$ (17)

where *N* represents the number of assessment levels and *L* represents the number of rules. $\beta_{i,k}$ represents the belief degree of the *k*th rule for the *i*th assessment result.

**Step 6:** Generate utility value

The final belief degree after aggregation can be expressed as:

$$S(x)=\left\{\left(D_{n},{\hat{\beta }}_{n}\right),n=1,\ldots ,N\right\}$$ (18)

Suppose that the utility of a single consequent *D*_*n*_ is represented as *u*_*n*_, and the expected utility of *S*(*x*) can be expressed as:

$$u\left(S(x)\right)=\sum_{n=1}^{N}u_{n}{\hat{\beta }}_{n}$$ (19)

### 3.2 MTS-BRB-R training process

**Fig 4:**
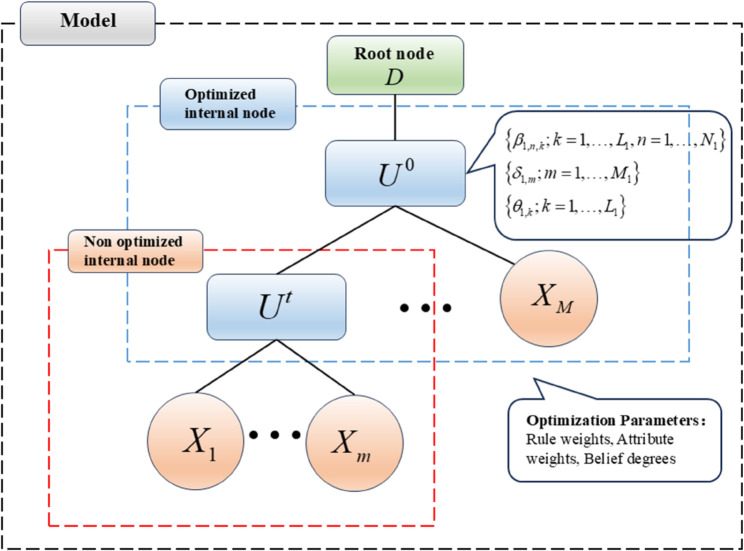
MTS-BRB-R optimization model.

Traditional BRB models rely on the relationship between the output results and the actual results to continuously optimize the parameters to achieve the effect of training the model. However, unlike the single-layer BRB model, since the H-BRB model takes the output of the sub-BRB model as the input of another BRB model, the sub-BRB model cannot optimize the model parameters directly on the basis of the actual results. Therefore, H-BRB adopts a training method in which the expert gives the parameters of the sub-BRB and trains only the BRB model that produces the final result, as shown in Fig 4. In this paper, the whole model is trained such that the expert gives the parameters of the BRB model of the internal node and trains the first internal node during training. For parameter optimization, we employ the Projected Covariance Matrix Adaptation Evolution Strategy (P-CMA-ES) algorithm to handle the constrained optimization problem in BRB training [26,27]. The parameter settings for the P-CMA-ES are provided in Table 3.

**Table 3:** Parameter settings of the P-CMA-ES.

Attribute	Symbol/Variable	Description and Value
Initial mean	*x*	Initial point of the population, user-defined input.
Population dimension	*N*	Number of decision variables, N=length(x).
Step size	*σ*	Initial coordinate-wise standard deviation, set to 0.08.
Fitness threshold	*stopfitness*	Termination when objective <10^−10^.
Maximum evaluations	*G*	Maximum number of generations, set to 400.
Upper bound	*ub*	Upper limit for each decision variable (input vector).
Lower bound	*lb*	Lower limit for each decision variable (input vector).
Equality constraints	$A_{eq},b_{eq}$	Constraint matrix and vector for the projection operator.

The training process is as follows:

Suppose that a set of input data is known $\left(x_{t},y_{t}\right)$, where $x_{t}=(x_{1,t},\ldots ,x_{m,t})$ (t=1,…,T) (m=1,…,M). *T* represents the number of input data. *M* represents the number of antecedent attributes. *y*_*t*_ is the observation in numerical form. For the input data *x*_*t*_, the distributed output generated via the ER is as Eq (20).

$${\hat{y}}_{t}=\left\{\left(D_{n},{\hat{\beta }}_{n}(t)\right),n=1,2,\ldots ,N\right\}$$ (20)

where ${\hat{\beta }}_{n}(t)$ denotes the belief degree generated by the ER algorithm with respect to the evaluation result *D*_*n*_. The average utility of the output can be obtained from the above equation as Eq (21).

$${\hat{y}}_{t}=\sum_{n=1}^{N}\mu (D_{n}){\hat{\beta }}_{n}(t)$$ (21)

where $\mu (D_{n})$ denotes the utility of the output portion of the rule. $\mu (D_{n})$ is determined by the expert.

The training process of MTS-BRB-R can be summarized as the desire to have the output of the highest-level BRB system ${\hat{y}}_{t}$ close to the possible approximation *y*_*t*_. Its optimization process can be expressed as Eq (22).

$$\min_{V}\left\{\frac{1}{T}\left(\sum_{t=1}^{T}{\left(y_{t}-{\hat{y}}_{t}\right)}^{2}\right)\right\}$$ (22)

where $V_{inner_{1}}={\left[\theta_{k},\delta_{i},\beta_{n,k},\mu (D_{n})\right]}^{T}$ denotes the parameter vector of *inner*_1_ in the MTS-BRB-R, k=1,2,…,L, i=1,2,…,M, and n=1,2,…,N. *L*, *M*, *N* represent the number of rules, the number of attributes, and the number of evaluation levels in *inner*_1_, respectively.

## 4 Case study

In this section, the prediction of the tailings silica content during iron ore flotation is used as an example to verify the validity of the model.

### 4.1 Experimental definitions

This paper uses publicly available datasets provided by the Kaggle platform. Kaggle is the world’s leading platform for data science and machine learning and is widely used in machine learning research and practice. The experimental data come from the Quality Prediction in a mining process dataset on the Kaggle platform (Kaggle, 2018). The dataset contains 22 parameters from March 2017–September 2017 of the iron ore concentration process in a flotation plant. The first column of the dataset indicates the time and date, the second and third columns represent the percentage of iron ore content and the percentage of silicon content before the iron ore slurry is fed into the flotation plant, and the ninth through twenty-second columns represent the process data during the flotation process. The specific selected dataset feature information is shown in Table 4.

**Table 4:** Physical significance of each parameter.

Attribute	Symbol	Description
*X*_1_	Iron Feed	Iron content of ore feed
*X*_2_	Silica Feed	Silicon content of ore feed
*X*_3_	Starch Flow	Starch content as an inhibitor
*X*_4_	Ore Pulp pH	PH value of slurry
*X*_5_	Ore Pulp Density	Slurry density
*X*_6_	Flotation Column 03 Air Flow	Air intake of flotation column 03
*X*_7_	Flotation Column 01 Level	The liquid level height of flotation column 01
*X*_8_	Flotation Column 02 Level	The liquid level height of flotation column 02
*X*_9_	Iron Concentrate	Iron content in concentrate
*X*_10_	Silica Concentrate	Silicon content in concentrate

Among them, the iron content of the ore feed (*X*_1_), the silicon content of the ore feed (*X*_2_), the starch content (*X*_3_), the pH value of the slurry (*X*_4_), the density of the slurry (*X*_5_), the air intake of the flotation column 03 (*X*_6_), the liquid surface height of flotation column 01 (*X*_7_), the liquid surface height of flotation column 02 (*X*_8_), and the iron content of the concentrate (*X*_9_) are taken as input characteristics. The concentrated silicon content (*X*_10_) is used as a prediction.

In this experiment, to ensure the objectivity and reliability of the model performance evaluation, a standard data division strategy is adopted, and the original dataset is divided at a ratio of 7:3.

Specifically, given the time-series nature of the flotation process data, we employed a chronological split to prevent data leakage: the first 70% of the data in temporal order are allocated to the training set, while the remaining 30% are used as an independent test set. This chronological partitioning ensures that the model is evaluated on future unseen data, preventing any temporal information leakage and providing a realistic assessment of the model’s predictive performance in practical applications.

Data standardization processing is a key step in eliminating the differences in feature scales and improving the stability of the algorithm. In this paper, there are features of different scales in the data, which, if directly input into the model, will lead to the distance-based algorithm being biased toward large-valued features owing to the difference in scales, reducing the prediction or clustering effect. Therefore, in this work, through Z-score standardization, each feature is converted to a distribution with a mean of 0 and a standard deviation of 1, which ensures that all the features participate in the calculation under the same scale, thus balancing their contributions to the model weights and enhancing the robustness and convergence efficiency of the algorithm.

To comprehensively assess the predictive performance and statistical reliability of the model, the following three evaluation indices are selected in this paper:

#### (1) Mean square error (MSE).

The MSE can effectively quantify the overall prediction deviation of the model by calculating the mean of the second-order moments between the predicted value and the true value, which is one of the core evaluation indices of the regression problem. Its calculation formula is as follows:

$$\text{mse}=\frac{1}{n}\sum_{i=1}^{n}(y_{i}-{\hat{y}}_{i}{)}^{2}$$ (23)

where *n* denotes the total number of samples. *y*_*i*_ denotes the true value of the *i*th sample. ${\hat{y}}_{i}$ denotes the predicted value of the *i*th sample.

#### (2) Coefficient of determination (*R*^2^).

*R*^2^ is a core metric used in statistics and machine learning to assess the goodness-of-fit of a regression model and is used to quantify the model’s ability to explain the variance of the target variable. Its calculation formula is as follows:

$$R^{2}=1-\frac{\sum_{i=1}^{n}(y_{i}-{\hat{y}}_{i}{)}^{2}}{\sum_{i=1}^{n}(y_{i}-\bar{y}{)}^{2}}$$ (24)

where *n* denotes the total number of samples. *y*_*i*_ denotes the true value of the *i*th sample. ${\hat{y}}_{i}$ denotes the predicted value of the *i*th sample. $\bar{y}$ denotes the mean of the true values.

#### (3) Core indicator decay rate (*PDR*).

*PDR* is used to quantify the degree of performance decay of the model under nonideal conditions such as noise interference, data distribution offset, or adversarial attack, reflecting the robustness of the model. It is an important evaluation index for evaluating the robustness of a model. Its definition is as follows:

$$PDR=\frac{\left(RMSE_{noise}-RMSE_{clean}\right)}{RMSE_{clean}}\times 100\%$$ (25)

where *RMSE*_*noise*_ and *RMSE*_*clean*_ denote the root mean square errors with and without added noise, respectively.

### 4.2 Modeling

The model construction is divided into two parts: Part A is the construction of the multilayer tree structure. Part B is the construction of the BRB model.

#### Part A: Constructing a multi-level tree structure.

This part describes the construction of a Multi-Level Tree Structure.

**Step 1:** Determine the root node and the first internal node

First, the prediction consequent attribute is determined, and the silicon content *X*_10_ is concentrated as the root node: *Root* = *X*_10_. Next, set the attribute *X*_10_ reference value as *SC*_*n*_, where n=(1,…,5), which takes the values shown in Table 5. Finally, the first internal node *U*^0^ is set as the set consisting of all input features, denoted as

$$U^{0}=\left\{X_{1},X_{2},X_{3},X_{4},X_{5},X_{6},X_{7},X_{8},X_{9}\right\}$$ (26)

**Table 5:** Reference values for silicon content.

SC	$SC_{1}$	$SC_{2}$	$SC_{3}$	$SC_{4}$	$SC_{5}$
Entirety BRB	0	1.87	3.1111	4.17	5.5001

**Step 2:** Calculate the AR value between features

According to the step-by-step formula shown in Part A of Sect 3.1, the AR values between all the features in the internal node *U*^0^ are computed to obtain the AR value table shown in Table 6.

**Table 6:** Correlation between attributes used for the tailings silica content prediction.

AR	$X_{1}$	$X_{2}$	$X_{3}$	$X_{4}$	$X_{5}$	$X_{6}$	$X_{7}$	$X_{8}$	$X_{9}$	sum(AR)
*X*_1_	1	0.985	0.409	0.413	0.360	0.288	0.388	0.408	0.566	4.817
*X*_2_	0.985	1	0.403	0.406	0.355	0.285	0.382	0.401	0.559	4.776
*X*_3_	0.409	0.403	1	0.990	0.816	0.829	0.962	0.986	0.623	7.019
*X*_4_	0.413	0.406	0.990	1	0.818	0.830	0.963	0.987	0.627	7.035
*X*_5_	0.360	0.355	0.816	0.818	1	0.676	0.792	0.814	0.544	6.174
*X*_6_	0.288	0.285	0.829	0.830	0.676	1	0.804	0.826	0.478	6.016
*X*_7_	0.388	0.382	0.962	0.963	0.792	0.804	1	0.959	0.598	6.849
*X*_8_	0.408	0.401	0.986	0.987	0.814	0.826	0.959	1	0.621	7.002
*X*_9_	0.566	0.559	0.623	0.627	0.544	0.478	0.598	0.621	1	5.616

**Step 3:** Clustering on the basis of the distance between features

The maximum number of clusters *ε* is set to 3, and the first three main factors are selected on the basis of the computed AR value with the following selection Eqs (27)–(29):

$$P_{1}=\arg\min_{X_{m}\in U^{0}}\left\{\sum_{X_{n}\in U^{0}}AR\left(X_{m},X_{n}\right)\right\}=X_{2}$$ (27)

$$P_{2}=\arg\min_{X_{m}\in U^{0}-\left\{P_{1}\right\}}\left\{\sum_{X_{n}\in \left\{P_{1}\right\}}AR\left(X_{m},X_{n}\right)\right\}=X_{1}$$ (28)

$$P_{3}=\arg\min_{X_{m}\in U^{0}-\left\{P_{1},P_{2}\right\}}\left\{\sum_{X_{n}\in \left\{P_{1},P_{2}\right\}}AR\left(X_{m},X_{n}\right)\right\}=X_{9}$$ (29)

*X*_1_, *X*_2_, and *X*_9_ are the three smallest features selected on the basis of the sum of the AR values, which serve as the initial three main elements, and the rest of the sets of elements are added to the set marked by the initial main elements according to the size of the AR values of the initial elements, for example:

$$AR(X_{4},X_{1})=0.41254,\;AR(X_{4},X_{2})=0.40621,\;AR(X_{4},X_{9})=0.62697,\;\;AR(X_{4},X_{9})>AR(X_{4},X_{1})>AR(X_{4},X_{2})$$ (30)

**Fig 5:**
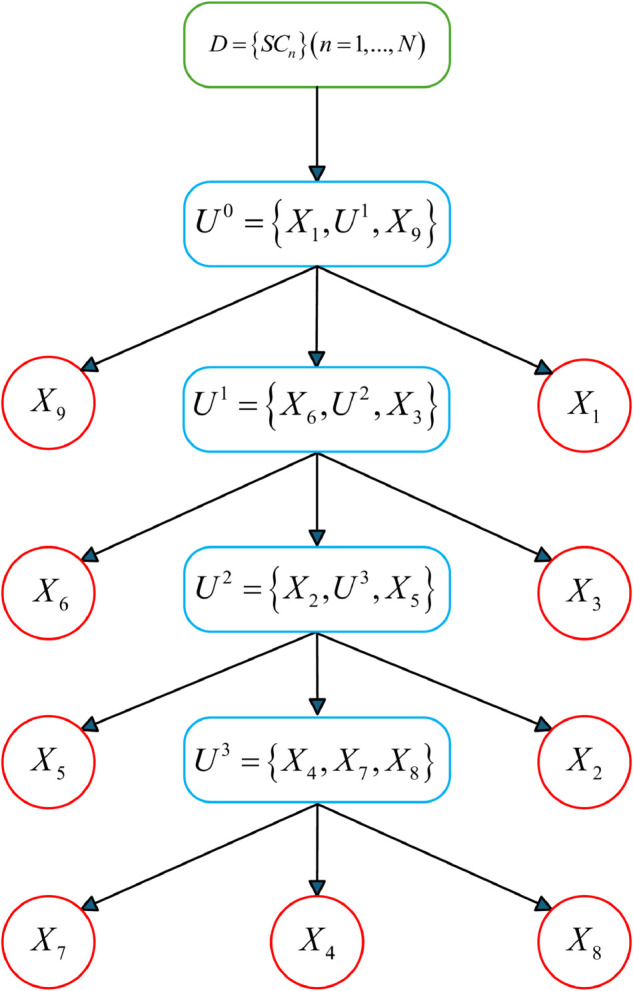
Final model structure.

According to the above equation, *X*_4_ should be added to the set {*P*_3_} where the pivot point *P*_3_ is located to form a new set $\left\{P_{3},X_{4}\right\}$. In addition, according to the above method, the remaining elements are added to the cluster where the main element is located, and the final formation of the result is shown in Fig 5.

#### Part B: Modeling the BRB.

This part describes the modeling of the Multi-Level Tree Structure.

**Step 1:** Calculate attribute reliability

According to Part B of Sect 3.1, only the attribute reliability of the first internal node *U*^0^ is calculated in this paper. The internal node *U*^0^ consists of attribute *X*_1_, internal node *U*^1^, and attribute *X*_9_. According to Eq (12), this study obtain that attribute *X*_1_ reliability *r*_1_ = 0.9809, attribute *X*_9_ reliability *r*_9_ = 0.8995, and internal node *U*^1^ reliability  = 1.0, where the tolerance *ψ* given by experts is 1.88.

**Step 2:** Build the model

After completing the attribute clustering, a total of three sub-BRBs and one prediction BRB are generated by assigning three reference values {Low,Medium,High} to each feature attribute and five reference points {VeryLow=0,Low=1.87,Medium=3.1111,High=4.17,VeryHigh=5.5001} to the consequent attribute *X*_10_ and, in particular, three reference values {Low=0.01,Medium=0.5,High=1.001} to the outputs of the sub-BRBs. Each BRB adopts a three-attribute structure, 27 rules are generated for each sub-BRB, and a total of 108 rules are generated.

In the MTS-BRB-R model for the prediction of the silica content in ore flotation concentrates, the belief rule for predicting the BRB can be described as Eq (31).

$$R_{k}:\text{If }(X_{1}\text{ is }A_{1}^{k})\wedge (X_{9}\text{ is }A_{9}^{k})\wedge (SubBRB\text{ is }A_{sub}^{k}),\;\;\text{Then predict result is }\{(SC_{1},\beta_{1,k}),(SC_{2},\beta_{2,k}),(SC_{3},\beta_{3,k}),(SC_{4},\beta_{4,k}),(SC_{5},\beta_{5,k})\}\;\;\text{with rule weight }\theta_{k},\text{ attribute weights }\delta_{1},\delta_{9},\delta_{sub}\;\;\text{and attribute reliabilities }r_{1},r_{9},r_{sub}$$ (31)

where $A_{1}^{k}$ and $A_{9}^{k}$ are the reference values of the two attributes; in particular, $A_{sub}^{k}$ is the reference value of the sub-BRB result. *SubBRB* represents the result of the sub-BRB output. Both attributes *X*_1_ and attributes *X*_9_ and *SubBRB* have three reference values. Thus, 27 combinations of the three attributes form the 27 rules for predicting the BRB in the MTS-BRB-R model. The initial parameters of the predicted BRB are given by the expert, as shown in Table 7.

**Table 7:** Rule base distribution for the initial model.

No.	Rule weight	Attribute			Consequent
		$X_{1}$	$X_{9}$	*SubBRB*	
1	0.2316	L	L	L	{0.0886 0.0957 0.2800 0.3290 0.2066}
2	0.3007	L	L	M	{0.0134 0.0824 0.0633 0.2271 0.6138}
3	0.1953	L	L	H	{0.1075 0.1494 0.1141 0.0583 0.5708}
4	0.3730	L	M	L	{0.1215 0.2264 0.1904 0.2335 0.2282}
5	0.1195	L	M	M	{0.0390 0.0083 0.0350 0.0889 0.8288}
6	0.1587	L	M	H	{0.4294 0.2141 0.2171 0.0514 0.0880}
7	0.1535	L	H	L	{0.0438 0.1961 0.1675 0.0585 0.5341}
8	0.1883	L	H	M	{0.0066 0.1025 0.1958 0.0701 0.6250}
9	0.3662	L	H	H	{0.0192 0.1092 0.0590 0.3120 0.5006}
10	0.1999	M	L	L	{0.2974 0.1323 0.2192 0.0479 0.3032}
11	0.0487	M	L	M	{0.1576 0.3420 0.4149 0.0667 0.0188}
12	0.1969	M	L	H	{0.2304 0.0612 0.1434 0.1813 0.3838}
13	0.4982	M	M	L	{0.1780 0.1989 0.3169 0.0811 0.2250}
14	0.1969	M	M	M	{0.2759 0.0400 0.3904 0.2239 0.0698}
15	0.0768	M	M	H	{0.0212 0.0637 0.2071 0.4339 0.2743}
16	0.0586	M	H	L	{0.5905 0.0638 0.0709 0.0391 0.2357}
17	0.1694	M	H	M	{0.1701 0.1065 0.3191 0.3346 0.0697}
18	0.3374	M	H	H	{0.0861 0.0417 0.1024 0.1735 0.5964}
19	0.1530	H	L	L	{0.0918 0.3162 0.1298 0.0315 0.4307}
20	0.1627	H	L	M	{0.2891 0.1678 0.2324 0.0998 0.2109}
21	0.0827	H	L	H	{0.3873 0.1869 0.0391 0.3158 0.0709}
22	0.2642	H	M	L	{0.0485 0.4076 0.3471 0.1669 0.0298}
23	0.4670	H	M	M	{0.7425 0.0569 0.0898 0.0440 0.0668}
24	0.0421	H	M	H	{0.1659 0.2611 0.4705 0.0261 0.0765}
25	0.2659	H	H	L	{0.1801 0.1785 0.2723 0.2982 0.0709}
26	0.0478	H	H	M	{0.3450 0.1735 0.3148 0.0667 0.1000}
27	0.1772	H	H	H	{0.3650 0.3727 0.0094 0.1998 0.0530}

### 4.3 Experimental analysis

#### (A) Analysis of model structure.

The complexity of the H-BRB model can be reflected in two aspects: the number of rules and the rational basis for constructing the model. As the number of rules increases, the model complexity increases. To make a more intuitive comparison, it is assumed that there are *M* antecedent attributes with 3 evaluation levels for each attribute, which leads to the construction of 4 types of BRBs, including traditional BRBs and MTS-BRBs with 2/3/4 subnodes, and the following figure represents the number of rules for these BRBs, where the number of subnodes indicates the maximum number of subnodes for each internal node.

**Fig 6:**
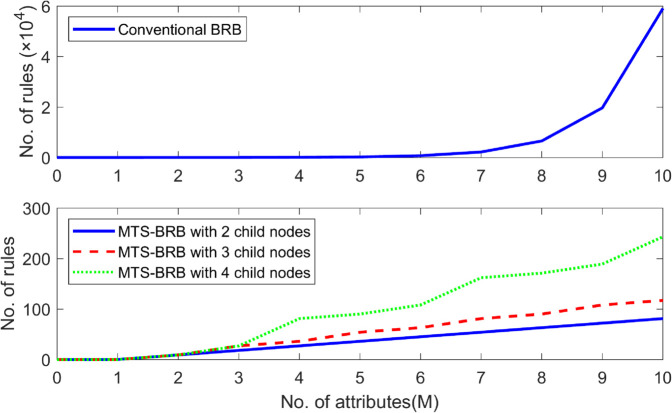
Number of different model rules.

Fig 6 clearly shows that the MTS-BRB approach results in approximately linear growth in model size with increasing number of attributes for different subnode configurations (2, 3, or 4 subnodes). A quantitative comparison shows that the number of rules required by the MTS-BRB system is significantly lower than that of the traditional BRB method. Specifically, when the number of attributes reaches 10, the traditional BRB system needs to generate 3^10^ = 59,049 rules, whereas only 81 (2-subnode), 117 (3-subnode), or 243 (4-subnode) rules are required when the MTS-BRB method is used, which are equivalent to 0.14%, 0.20%, and 0.41% of the number of rules of the traditional method, respectively. This comparison fully demonstrates the efficiency of the MTS-BRB method in controlling the number of rules, which effectively reduces the model complexity through the hierarchical tree structure. In addition, compared with the traditional H-BRB, which relies on manual organization construction, the MTS-BRB has greater rationality because the MTS-BRB organizes the model structure via a computational method that is based on entropy and mutual information.

#### (B) Model robustness analysis.

The robustness of the model is reflected mainly in the performance of the model in the presence of noise interference. Its evaluation index PDR is defined by Eq (25). where PDR represents the performance change caused by noise, and the larger the value is, the weaker the robustness of the model. In this work, three scenarios are designed to verify the model robustness: (1) adding Gaussian noise (2) adding impulse noise (3) combining Gaussian noise and impulse noise. Each scenario is designed with three interference strengths: weak, medium, and strong. The PDR changes of the global noise and characteristic interference are tested separately. In this study, all models, trained on clean data, were evaluated on noisy test sets to simulate online inference. The variation in the PDR is analyzed in the following two figures. The scenarios are designed as shown in Table 8.

**Table 8:** Noise settings for different scenarios.

Scene	Noise Strength	*GN*	*IN*
Gaussian Noise	Low	σ=0.1	N/A
	Medium	σ=0.2	N/A
	High	σ=0.3	N/A
Impulse Noise	Low	N/A	p=5%
	Medium	N/A	p=10%
	High	N/A	p=15%
Gaussian and Impulse Noise	Low	σ=0.1	p=5%
	Medium	σ=0.2	p=10%
	High	σ=0.3	p=15%

**Fig 7:**
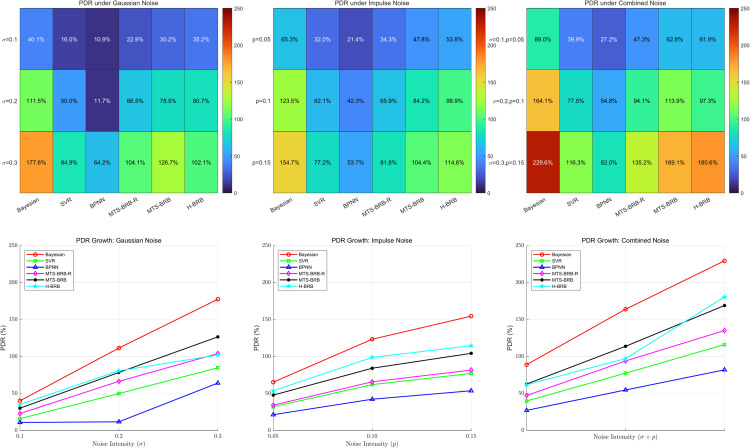
Global noise.

**Fig 8:**
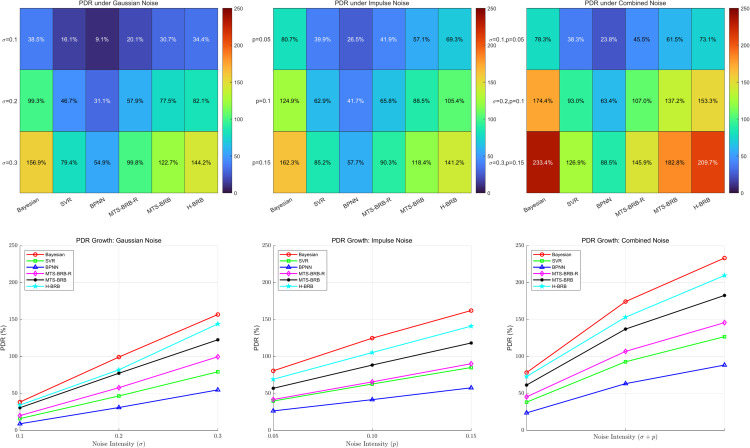
Partial noise.

In this paper, the effects of global noise and feature noise on model performance are evaluated separately. As shown in Figs 7 and 8, both global noise and feature noise lead to a systematic increase in PDR for all the models. Notably, MTS-BRB-R has superior stability in both noise scenarios, but its advantage is more prominent in feature noise.

According to the experimental data, the performance degradation rates (PDRs) of all the tested models significantly monotonically increased with increasing noise intensity (*σ*/p). This linear increasing relationship indicates a significant positive correlation between noise intensity and model performance degradation. Among the six compared models, the BPNN model exhibits the best noise immunity performance, with its PDR value remaining the lowest at all noise levels. Notably, among the BRB series of models, the MTS-BRB-R model exhibits superior robustness. The experimental data show that under the influence of moderate global noise (*σ* = 0.2), the PDR of the MTS-BRB-R (66.5%) is reduced by approximately 12% and 14.2% compared with those of the MTS-BRB and H-BRB, respectively. As shown in Figs 7 and 8, the PDR growth rate of MTS-BRB-R is significantly lower than that of H-BRB and MTS-BRB, confirming its better noise stability.

The superior performance of MTS-BRB-R may stem from the introduction of attribute reliability, which is designed to effectively suppress the propagation of noise in the inference process, thus maintaining greater stability. In contrast, the standard MTS-BRB and H-BRB models are more sensitive to noise disturbances and exhibit more substantial performance degradation, especially in high-noise environments.

#### (C) Analysis of model effects.

After the MTS-BRB-R model was built, a total of 2091 data points per hour in March from the mining flotation dataset were used as training data, and the test set was selected and normalized with data per hour in April. To illustrate the effectiveness of the MTS-BRB-R, in this paper, the MTS-BRB-R model is compared with other models.

The compared models are divided into two main categories: other BRB models and machine learning models. The BRB models include the BRB-PCA model constructed using data after PCA dimensionality reduction and the general hierarchical H-BRB and MTS-BRB models. Among them, PCA is a classical linear dimensionality reduction method based on orthogonal transformation, whose core idea is to preserve the maximum variance information in the data by projecting the high-dimensional data into a low-dimensional subspace spanned by principal components. The BRB-PCA model selects two principal components as inputs to construct the model. For H-BRBs, the model is constructed by randomly assigning three features to sub-BRBs. The MTS-BRB model constructs the model results in an ordered manner by calculating the AR values. Machine learning models include Bayesian regression, SVR, and BPNN. The performance analysis of each model is shown in Table 9.

**Table 9:** Performance comparison of different models.

Metric	BR	SVR	BPNN	BRB-PCA	H-BRB	MTS-BRB	MTS-BRB-R
*RMSE*	0.49	0.5084	0.6447	1.7239	0.5365	0.4974	0.4558
*MAE*	0.38	0.3821	0.5262	1.4791	0.4201	0.3776	0.3518
*R* ^2^	0.85	0.8415	0.7452	–0.8661	0.8074	0.8345	0.8611

**Fig 9:**
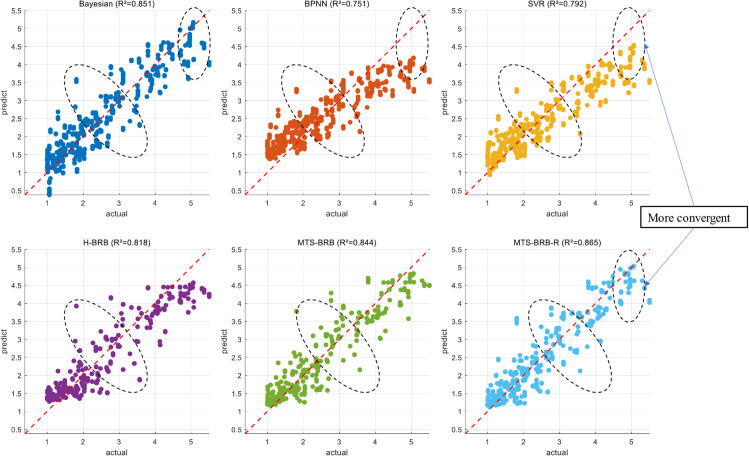
Model performance.

In the diagonal scatter plot in Fig 9, ideally, all points should fall on the diagonal line (y = x), and deviations from the diagonal line indicate prediction errors. The points above the diagonal line indicate that the predictions are systematically high, and the points below the focus line indicate that the predictions are systematically low. As seen from the scatterplot, most models perform better in the region of small values (<2). Among them, the H-BRB, MTS-BRB, and MTS-BRB-R models show a trumpet trend distribution in the small-value region, which indicates that the error shows an increasing trend with increasing value. In the median region, the three models show a more regular distribution of outlier points, among which the degree of the outlier points of the MTS-BRB-R model is weaker than that of the remaining two models, and on the whole, the outlier points show a more regular distribution with the improvement of the model. With the improvement of the model, the outliers show a convergence trend; in the large value region, H-BRB shows a more obvious systematic underprediction, and with the improvement of the model, the underprediction problem shows an obvious improvement trend. A horizontal comparison of the MTS-BRB-R model reveals that the advantage of the MTS-BRB-R model is reflected mainly in the small-value region, which has a more favorable convergence trend, and in the large-value region, which overcomes the problem of systematic underprediction. Combining the horizontal and vertical comparisons, it can be proven that the MTS-BRB-R model has a superior modeling effect.

Combining the model robustness and model accuracy, the MTS-BRB-R model is more robust and accurate than the H-BRB model is. The Bayesian, SVR, and BPNN models have superior robustness, but their robustness is based on a lower initial accuracy. The MTS-BRB-R model is more robust than the other BRB models are; thus, it is reasonable and effective to solve the BRB multiattribute input problem with the MTS structure.

The superior performance of the proposed MTS-BRB-R model can be attributed to its unique structural and mechanistic advantages over both traditional BRB models and data-driven neural networks. Compared to traditional BRB, the multilayer tree structure effectively alleviates the rule explosion problem by decomposing the complex high-dimensional input space into manageable sub-modules, each focusing on a subset of attributes with high mutual correlation. This not only reduces model complexity but also enhances interpretability by providing a clear hierarchical reasoning path.

When compared to neural network models (e.g., BPNN, SVR), the MTS-BRB-R combines the strengths of both expert knowledge and data-driven learning. While neural networks often act as “black boxes" with limited interpretability, our model maintains transparent decision-making through its belief rule structure. The incorporation of attribute reliability further enhances robustness against noisy industrial data, which is a common challenge in flotation process monitoring. This hybrid modeling approach allows the MTS-BRB-R to achieve both high accuracy and reliability, making it particularly suitable for complex industrial processes where both performance and interpretability are crucial.

#### (D) Sensitivity analysis of tolerance parameter *ψ.*

The calculation of attribute reliability depends on the tolerance parameter *ψ* provided by domain experts. To verify the robustness of the model against variations in this parameter, we conducted a sensitivity analysis by testing *ψ* values ranging from 0.5 to 3.0. The performance metrics under different *ψ* values are summarized in Table 10.

**Table 10:** Sensitivity analysis of tolerance parameter ψ.

ψ value	RMSE	MAE	R^2^
0.50	0.4612	0.3589	0.8423
0.75	0.4578	0.3552	0.8578
1.00	0.4563	0.3531	0.8592
1.25	0.4559	0.3520	0.8601
1.50	0.4557	0.3515	0.8605
1.75	0.4558	0.3516	0.8604
1.88	0.4558	0.3518	0.8611
2.00	0.4560	0.3521	0.8602
2.25	0.4565	0.3528	0.8594
2.50	0.4572	0.3536	0.8583
2.75	0.4581	0.3545	0.8571
3.00	0.4593	0.3557	0.8556

The results demonstrate that the MTS-BRB-R model maintains stable performance across a wide range of *ψ* values (1.25 to 2.25), with RMSE variations within 0.2

## 5 Conclusion

Indicator prediction in the flotation process is a complex structured problem. Traditional BRB methods often lead to rule explosion when handling multiple parameters, as the number of rules grows via the Cartesian product of input references. Existing hierarchical models also largely rely on empirical segmentation without systematic construction frameworks. To overcome these issues, this paper proposes an MTS-BRB-R model based on a multilayer tree structure, which incorporates attribute reliability to enhance robustness and prediction accuracy.

The MTS-BRB-R model is particularly suited for complex systems like flotation with correlated inputs and local feature noise. It enables structured decomposition and reliability-aware inference. The experimental results demonstrate that the MTS-BRB-R model achieves superior performance compared to both traditional BRB and neural network approaches, owing to its effective integration of structural optimization through multilayer tree decomposition, enhanced robustness via attribute reliability mechanisms, and maintained interpretability through transparent belief rule reasoning.

However, limitations remain. Currently, only antecedent attribute reliability is considered, while sub-BRB outputs are assumed fully reliable, potentially affecting credibility assessment. The clustering of element sets still depends on subjective definitions, influencing structural optimization. Moreover, while competent under local noise, the model’s robustness against global systematic noise requires improvement. The mechanism through which attribute reliability affects overall model reliability also needs deeper investigation. Future work will focus on: (1) improving reliability computation for sub-BRBs; (2) developing adaptive clustering to automate element set partitioning; (3) enhancing robustness under global noise; (4) establishing a comprehensive reliability evaluation framework linking attribute-level and model-level performance.
